# Kissing G Domains of MnmE Monitored by X-Ray Crystallography and Pulse Electron Paramagnetic Resonance Spectroscopy

**DOI:** 10.1371/journal.pbio.1000212

**Published:** 2009-10-06

**Authors:** Simon Meyer, Sabine Böhme, André Krüger, Heinz-Jürgen Steinhoff, Johann P. Klare, Alfred Wittinghofer

**Affiliations:** 1 Department of Structural Biology, Max-Planck-Institute of Molecular Physiology, Dortmund, Germany; 2 Department of Physics, University of Osnabrück, Osnabrück, Germany; Brandeis University, United States of America

## Abstract

MnmE, which is involved in the modification of the wobble position of certain tRNAs, belongs to the expanding class of G proteins activated by nucleotide-dependent dimerization (GADs). Previous models suggested the protein to be a multidomain protein whose G domains contact each other in a nucleotide dependent manner. Here we employ a combined approach of X-ray crystallography and pulse electron paramagnetic resonance (EPR) spectroscopy to show that large domain movements are coupled to the G protein cycle of MnmE. The X-ray structures show MnmE to be a constitutive homodimer where the highly mobile G domains face each other in various orientations but are not in close contact as suggested by the GDP-AlF_x_ structure of the isolated domains. Distance measurements by pulse double electron-electron resonance (DEER) spectroscopy show that the G domains adopt an open conformation in the nucleotide free/GDP-bound and an open/closed two-state equilibrium in the GTP-bound state, with maximal distance variations of 18 Å. With GDP and AlF_x_, which mimic the transition state of the phosphoryl transfer reaction, only the closed conformation is observed. Dimerization of the active sites with GDP-AlF_x_ requires the presence of specific monovalent cations, thus reflecting the requirements for the GTPase reaction of MnmE. Our results directly demonstrate the nature of the conformational changes MnmE was previously suggested to undergo during its GTPase cycle. They show the nucleotide-dependent dynamic movements of the G domains around two swivel positions relative to the rest of the protein, and they are of crucial importance for understanding the mechanistic principles of this GAD.

## Abstract 2

The authors of this research article demonstrate the nature of the conformational changes MnmE was previously suggested to undergo during its GTPase cycle, and show the nucleotide-dependent dynamic movements of the G domains around two swivel positions relative to the rest of the protein. These movements are of crucial importance for understanding the mechanistic principles of this GAD.

## Author Summary

MnmE is an evolutionary conserved G protein that is involved in modification of the wobble U position of certain tRNAs to suppress translational wobbling. Despite high homology between its G domain and the small G protein Ras, MnmE displays entirely different regulatory properties to that of many molecular switch-type G proteins of the Ras superfamily, as its GTPase is activated by nucleotide-dependent homodimerization across the nucleotide-binding site. Here we explore the unusual G domain cycle of the MnmE protein by combining X-ray crystallography with pulse electron paramagnetic resonance (EPR) spectroscopy, which enables distance determinations between spin markers introduced at specific sites within the G domain. We determined the structures of the full-length MnmE dimer in the diphosphate and triphosphate states, which represent distinct steps of the G domain cycle, and demonstrate that the G domain cycle of MnmE comprises large conformational changes and domain movements of up to 18 Å, in which the G domains of the dimeric protein traverse from a GDP-bound open state through an open/closed equilibrium in the triphosphate state to a closed conformation in the transition state, so as to assemble the catalytic machinery.

## Introduction

Cells devote substantial biosynthetic effort and resources to posttranscriptional modification of tRNAs [1]. A frequent feature of tRNAs in all domains of life are modified nucleosides in the anticodon region and especially at the wobble position (position 34) [2], which prestructure the anticodon domain to insure correct codon binding during translation [3]. MnmE is an evolutionary conserved G protein found in bacteria, fungi, and humans, which together with the protein GidA catalyzes the formation of a carboxymethylaminomethyl-group (cmnm) at the 5 position of the wobble uridine (U34) of tRNAs reading 2-fold degenerated codons ending with A or G, i.e., tRNA_Arg_(UCU), tRNA_Gln_(UUG), tRNA_Glu_(UUC), tRNA_Leu_(UAA), and tRNA_Lys_(UUU) [4]–[6]. This modification (cmnm^5^U34) together with a thiolation at the 2 position favours the interaction with A and G, but suppresses base-pairing with C and U [3],[7]–[10]. By controlling rare codon recognition and reading frame maintenance, hypermodified U34 moreover plays a regulatory role in gene expression [11]. Eucaryotic homologues of MnmE and GidA (termed MSS1 and Mto1, respectively, in yeast) are targeted to mitochondria [12],[13], and the human homologues (termed hGTPBP3 and Mto1, respectively) have been implicated in the development of severe mitochondrial myopathies such as MERRF (myoclenic epilepsy ragged red fibres), MELAS (mitochondrial encephalomyopathy lactic acidosis stroke), and nonsyndromic deafness [14]–[18].

**Figure 1:**
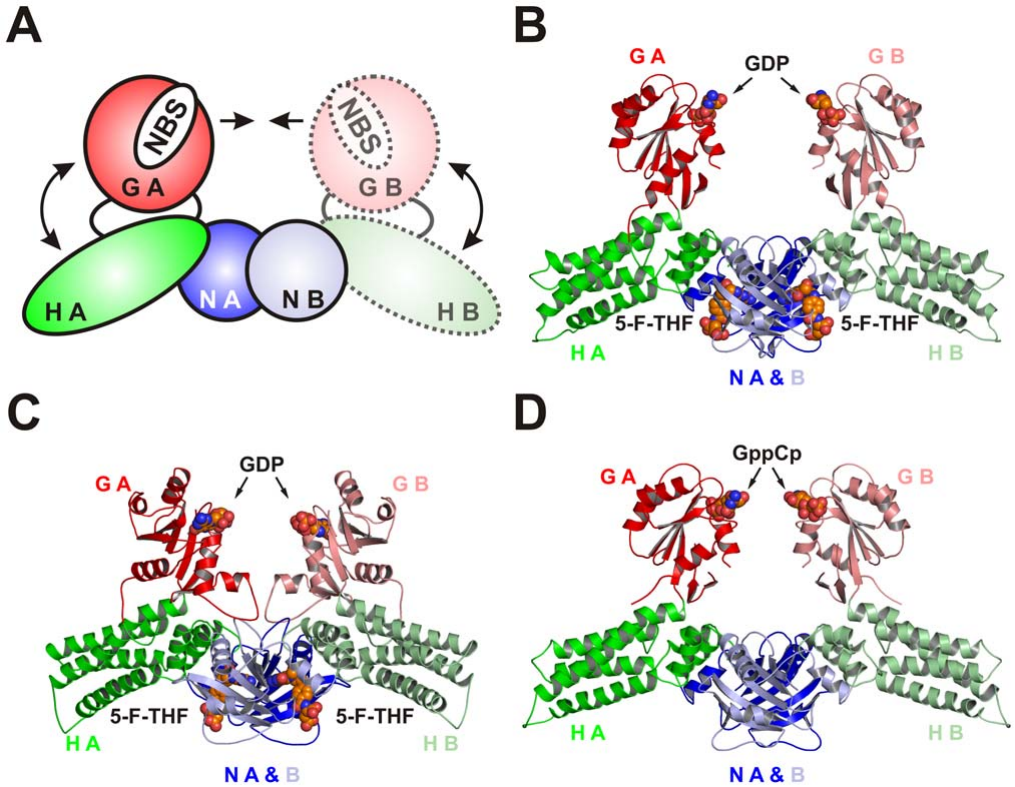
X-ray structures of full-length MnmE dimers. (A) Model of dimeric MnmE obtained from the partial structure of nucleotide-free MnmE from *T. maritima*, where only the N-terminal domain (NB), but not the helical (HB), or G domain (GB) of molecule B were present in the crystal. The model was obtained by superimposition of molecule A on the N domain of B and the expected positions of the nucleotide binding sites (denoted as NBS) in this model are indicated. (B–D) Ribbon models of X-ray structures of CtMnmE·GDP (B), No MnmE·GDP (C), and Ct MnmE·GppCp (dimer a) (D), with colors of the N, H, and G domains as indicated, and the protomers A and B.

The crystal structure of MnmE from *Thermotoga maritima* reveals a three-domain protein consisting of an N-terminal tetrahydrofolate-binding domain, a central helical domain, and a canonical Ras-like G domain inserted into the helical domain [19]. The asymmetric unit of these crystals contained one MnmE molecule and the N-terminal domain of a second proteolysed MnmE chain interacting with the N-terminal domain of the first molecule, suggesting that MnmE is a dimer in solution (Figure 1A) [19]. By superposition of the first MnmE chain on the second N-terminal domain a model for the full-length homodimer was generated in which the two G domains face each other with a distance of almost 50 Å between the two P-loops (Figure 1A) [19].

In contrast to Ras-like small G proteins that require a guanine nucleotide exchange factor (GEF) protein to drive the nucleotide exchange and a GTPase activating protein (GAP) to stimulate hydrolysis [20],[21], MnmE displays lower affinities towards nucleotides and a higher intrinsic K^+^-stimulated GTP hydrolysis [19],[22]–[24]. A G domain dimerization across the nucleotide binding site has been proposed on the basis of biochemical data and the crystal structure of the isolated MnmE G domains in complex with GDP-aluminium tri- or tetrafluoride (AlF_x_) (a mimic of the transition state of GTP hydrolysis [25]) [22]. The G domains dimerize via their switch regions to position an invariant Glu-residue (E282) for optimal orientation of a water molecule for the nucleophilic attack of the γ-phosphate group [22]. Dimerization stabilizes a highly conserved loop in switch I, the so-called K-loop, to coordinate K^+^ in a position analogous to the positive charge of the arginine finger in the Ras-RasGAP system. This explains why K^+^ is required both for the GTPase stimulation and for G domain dimerization [22]. On the basis of the common feature that the G domain cycle is regulated by homodimerization, MnmE has been categorized as G protein activated by nucleotide-dependent dimerization (GAD) [26], together with the signal recognition particle (SRP) and its receptor (SR) [27],[28], the regulator of Ni insertion into hydrogenases HypB [29], the dynamins [30], the human guanylate binding protein hGBP1 [31], the chloroplast import receptors Toc33/34 [32],[33], the septins [34], and the Roc-COR tandem found to be mutated in Parkinson disease [35]. It has been postulated that nucleotide-dependent G domain dimerization activates the GTPase and the distinct biological functions of these proteins, although the mechanisms of coupling G domain dimerization to biological function within this class are diverse and incompletely understood [26].

So far, neither the structural model of the full-length MnmE dimer nor dimerization of the G domains in the context of the full-length dimer have been proven directly. With the architecture of the proposed dimer model, dimerization of the G domains would require large domain movements suggesting that large conformational rearrangements of the protein are coupled to its GTPase cycle [22]. Here we study these GTPase-coupled rearrangements by trapping the protein in various steps of its GTPase cycle by X-ray crystallography and pulse double electron-electron resonance (DEER) spectroscopy in combination with site-directed spin labeling [36]–[38]. The distance distributions obtained for spin labeled sites in the G domains of MnmE allow us to characterize the G domain movements during the GTPase cycle of MnmE.

## Results

### Crystal Structures of Full Length MnmE Bound to GDP and GppCp

Various MnmE homologous have been screened for crystallization conditions in the presence of GDP, GDP-AlF_x_ and guanosine-5′-(β,γ-methylene)triphosphate (GppCp), and K^+^ and were found to crystallize readily in diverse conditions, but only in three cases—*Chlorobium tepidum* MnmE (CtMnmE) in the presence of K^+^, GDP, or GDP-AlF_x_; *Nostoc* MnmE (NoMnmE) in the presence of K^+^, GDP, or GDP-AlF_x_; and CtMnmE in the presence of K^+^ and GppCp-crystals with sufficient diffraction quality were obtained. In the case of CtMnmE, a polyethylene glycol (PEG) 6000/NaCl-condition produced diffraction quality crystals in the presence of GPD and GDP-AlF_x_. Crystals had the same unit cell parameters and the same space group and are thus isomorphous. NoMnmE crystals with sufficient diffraction were obtained in a PEG 550 monomethyl ether (MME) condition. As with CtMnmE, crystals obtained in the presence of GDP-AlF_x_ or GDP were isomorphous. Structure determination showed in both cases that the crystals contained the GDP-bound form of MnmE, despite the presence of AlF_x_. Quality of crystals grown in the presence of GDP-AlF_x_ were somewhat better, hence their datasets were used for structure determination.

CtMnmE·GDP and NoMnmE·GDP (grown in presence of AlF_x_) crystallized in the space groups I4(1)22 and P4(3)2(1)2, respectively, each with one full length protomer in the asymmetric unit. In both cases homodimers are formed via crystallographic symmetry by means of the N-terminal domains (Figure 1B and 1C). Apart from the location of G domains, the structure is very similar to the dimer model proposed for nucleotide-free MnmE (Figure 1A) [19]. Strikingly, two molecules of 5-formyl-tetrahydrofolate (5-F-THF) were identified in the structure of NoMnmE·GDP, which were apparently copurified from the bacterial expression system (Figure S1A). This suggests a high affinity for 5-F-THF and supports the recently proposed enzymatic mechanism whereby the C1 group of the cmnm modification is donated by THF [19],[39]. The cofactor is bound as previously described for the complex prepared in situ [19], with two folate binding sites within the dimer interface of the N-terminal domains. CtMnmE·GDP crystals were incubated with a 5-F-THF-containing cryoprotectant prior to data collection and in the crystal structure 5-F-THF is found in identical positions as in the NoMnmE·GDP-dimer (Figure S1B) and in the TmMnmE-dimer.

In the case of CtMnmE·GppCp, the crystallographic asymmetric unit contained three protomers (chains A, B, C). Molecules B and C form a dimer within the asymmetric unit, while protomer A forms a dimer with its crystallographic symmetry mate (shown in Figure 1D). No density is found for the G domain of molecule C, but crystals applied on an SDS-page confirmed an intact protein (unpublished data). Thus two dimeric structures of CtMnmE·GppCp were analyzed, i.e., the dimer generated by protomer A and a symmetry related chain A (termed “dimer A”) and the dimer generated by protomer B and a second protomer B docked onto chain C (termed “dimer B”).

The overall homodimer architecture found in the three structures resembles the proposed model obtained from a partial dimer (Figure 1A), with the G domains facing each other with their nucleotide binding sites (Figure 1B–1D). However, even though triphosphate analogues such as GppCp or AlF_x_ and GDP were used in the crystallization trials, the G domains were separated from each other by large distances. They do not display any structural contacts between each other nor to the N-terminal or helical domains. In all the structures, nucleotides are far apart from each other, with distances of 38 to 56 Å between the first P-loop glycines' Cα atom (GxxxxGKS motif).

### The Mobile G Domains

In each structure, the G domain adapts the canonical Ras-fold with either both switch regions (CtMnmE·GDP, CtMnmE·GppCp) or switch II (NoMnmE·GDP) disordered and thus not resolved. Nucleotides are bound in a way typical for Ras-like G domains (Figure S1C–S1E). In CtMnmE·GDP however, no Mg^2+^ is coordinated to the phosphates, and switch I-contacts to GDP are absent (Figure S1C). In NoMnmE·GDP, two Zn^2+^ atoms from the crystallisation condition, localized by their anomalous signal, are coordinated to the G domain. One of these is coordinated to helix Gα4 and is involved in crystal contacts (see below), the other occupies the usual Mg^2+^-binding site at the β-phosphate of GDP (Figure S1D). As Switch I is resolved, but does not contact the bound GDP and since there is no indication for a physiological role of Zn^2+^, we consider this to be a crystallographic artefact also observed in the nucleotide binding pockets of other small G proteins [40].

For conventional G proteins regulated by GAPs [20] as well as for G proteins activated by dimerization [26], AlF_x_-in the γ-phosphate binding site mimics the transition state of the phosphor transfer reaction and is considered the litmus test for correct assembly of the active site. In the case of MnmE, this is thought to be achieved by dimerization and close juxtaposition of the two G domains across the nucleotide binding site, as observed for the isolated G domains [22]. Although both GDP-bound structures have been obtained using GDP and AlF_x_ in the crystallization trials, no electron density for AlF_x_ could be observed (Figure S1C and S1D). One would thus conclude that close contact between the G domains is not possible in the full-length protein or that the G domains are too mobile for fixation in the crystal and/or that the crystal lattice forces do not allow the close state to occur.

Another possibility would be that crystallisation conditions with high concentrations of precipitants inhibit formation of the closed state of the G domains. Indeed we can show by a previously established fluorometric assay, by which an increase of the fluorescence of 2′-/3′-O-(N′-methylanthraniloyl)-GDP (mGDP) bound to MnmE upon addition of AlF_x_ in the presence of K^+^ is attributed to G domain dimerization [22], that in the presence of any of the precipitants used for crystallisation, dimerization of the G domains is severely inhibited in the full length protein (Figure S2). This explains why despite the presence of AlF_x_ in the crystallisation trials only the GDP-bound conformations are found. In the crystals, the G domains are thus trapped in an open state that does not allow tight binding of AlF_x_, into the γ-phosphate binding site.

**Figure 2:**
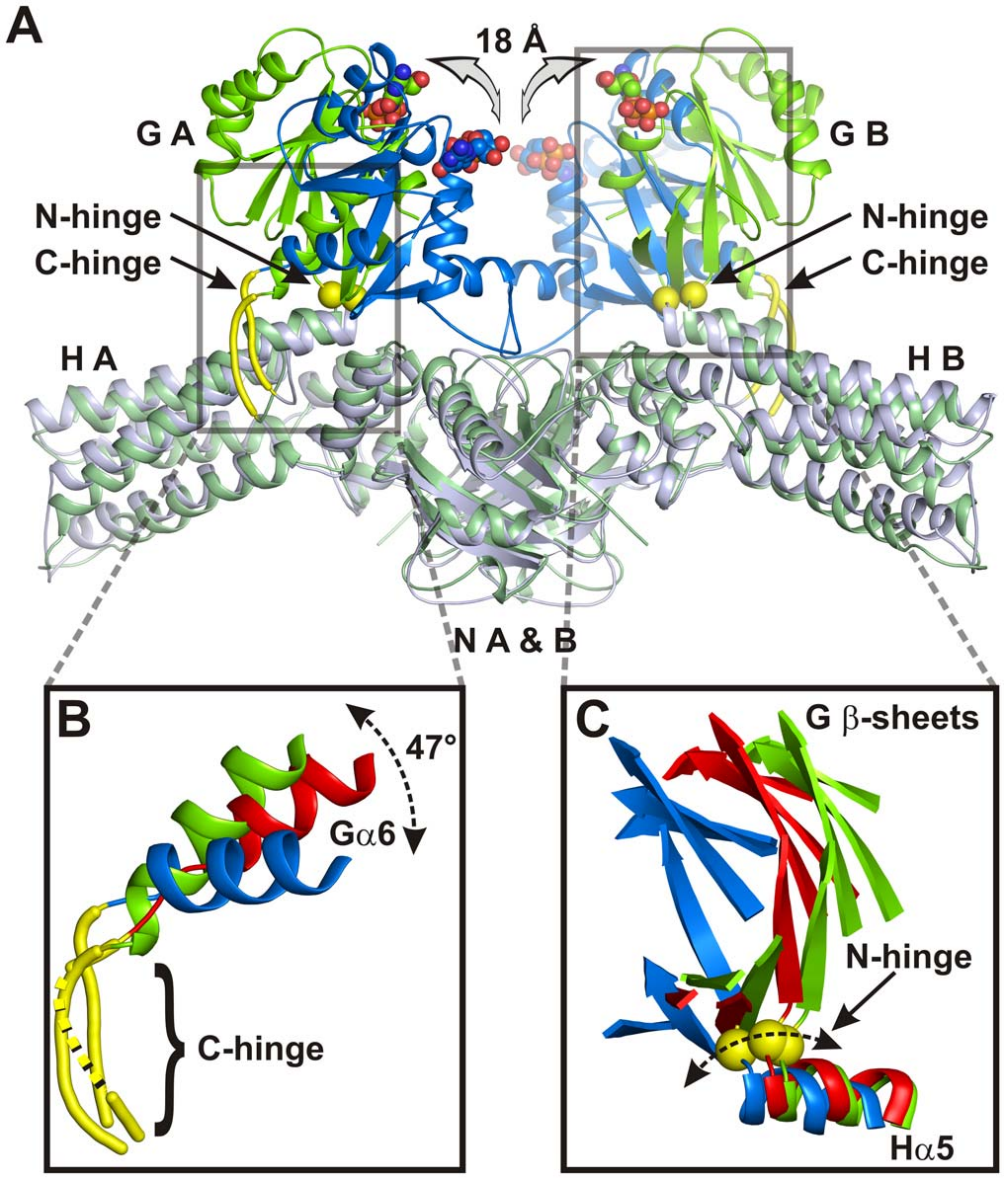
Orientations of the G domain. (A) Superimposition of CtMnmE·GDP (green) and NoMnmE•GDP (blue) dimers (displayed as ribbon models) via the N-terminal and helical domains with domains labeled as in Figure 1, highlighting the G domains, the relative movements of the nucleotides (displayed as spherical models), and the N- (yellow spheres) and C-hinge (yellow tubes), shown in detail in (B, C). (B, C) Superimposition of the C-hinge (B) and the N-hinge adjacent to helix Hα5 of the H domain (C) of the Ct and NoMnmE structures (coloring as in [A]) together with the corresponding parts of CtMnmE·GppCp, (chain A, red), highlighting the relative movements of the last helix of the G domain, Gα5 (B), and of the G domain β-sheet (C). The part of the C-hinge in CtMnmE·GppCp not resolved in the X-ray structure is depicted as dashed yellow line (B).

Superposition of the five available homodimer structures (CtMnmE·GDP, NoMnmE·GDP, CtMnmE·GppCp dimers of molecules A and B, *T. maritima* MnmE dimer model, generated with pdb 1XZP) reveals that the N-terminal domains align quite well and only minor displacements are present for the helical domains (Figures 2A and S3; Table 1). Strikingly, the superposition shows large rotational and translational displacements of the G domains (Figures 2A–2C and S3), which are reflected in their higher root mean square deviation (RMSD) values (Table 1) leading to separation of nucleotide binding sites between, for instance, CtMnmE·GDP and NoMnmE·GDP by 18 Å (Cα-Cα distance of the first P-loop glycines) (Figure 2A). This becomes clearly visible in the displacement of the G domain β-sheets and of helix Gα6 (Figure 2B and 2C). A video generated from the five homodimer structures makes the drastic displacements of the G domains evident and highlights the dynamic character of the G domains (Video S1).

**Table 1:** Average RMSD of each N-terminal domain, helical domain, and G domain from a superposition of the five MnmE structures (CtMnmE·GDP, CtMnmE·GppCp A and B, *T. maritima* MnmE [pdb 1XZP], NoMnmE·GDP) with NoMnmE·GDP as reference structure.

Average RMSD/Å to NoMnmE·GPD			
Domain	N-Terminal Domain	Helical Domain	G Domain
CtMnmE·GDP	1.37	1.79	9.47
CtMnmE·GppCp, chain A	1.04	1.64	7.62
CtMnmE·GppCp, chain B	1.28	2.31	9.84
*T. maritima* MnmE	1.59	2.61	6.53

The different orientations indicate that the G domains are highly flexible with regard to the rest of the protein probably due to the rather loose connections between G and helical domains. A conserved glycine residue is situated between helix Hα5 and the first strand of the G domain β-sheet (Figure 2C), which because of its higher conformational freedom could function as a hinge (“N-hinge”). A second hinge point (“C-hinge”) is where a not-well-ordered loop attaches the C-terminal end of the G domain after Gα6 to the helical domain (Figure 2B). The angle by which Gα6 is shifted spans up to 47°. In the crystal structure of CtMnmE·GppCp this loop region is not resolved underlining its high flexibility.

Although crystals grew under many more conditions, crystals diffracting to reasonable resolution were only obtained in the cases reported here. This result is most likely due to the fact that in these cases, crystal contacts trap the G domains in defined orientations (Figure S4), whereas in the weakly diffracting crystals the G domains are only loosely packed causing lattice disorder. The G domains in the CtMnmE·GDP and NoMnmE·GDP structures pack against symmetry mates with contact areas of 376 Å^2^ and 488 Å^2^. In NoMnmE·GDP a Zn^2+^-ion tightly links the G domain to symmetry mates (Figure S4A), while in CtMnmE·GDP the G domains fix each other by a toothing upside-down arrangement (Figure S4B). Crystal contacts of G domains A and B in the CtMnmE·GppCp structure comprise areas of 845 Å^2^ and 987 Å^2^, respectively. Docking the G domain of molecule B (or A) into the asymmetric unit of the CtMnmE·GppCp structure to the position expected for the G domain of molecule C would create a much smaller hypothetical crystal contact area of only 18 Å^2^ (or 131 Å^2^). Thus we would expect that the G domain of molecule C is present in the crystal but, due to its high mobility and absence of sufficient crystal contacts, is not visible in the electron density map. This is similar to the recent structure of the Roco protein, which is also a GAD protein. There, the second G domain of the constitutive dimer is present in the crystal but can not be identified in the electron density map [35].

### G Domain Mobility Measured by DEER

**Figure 3:**
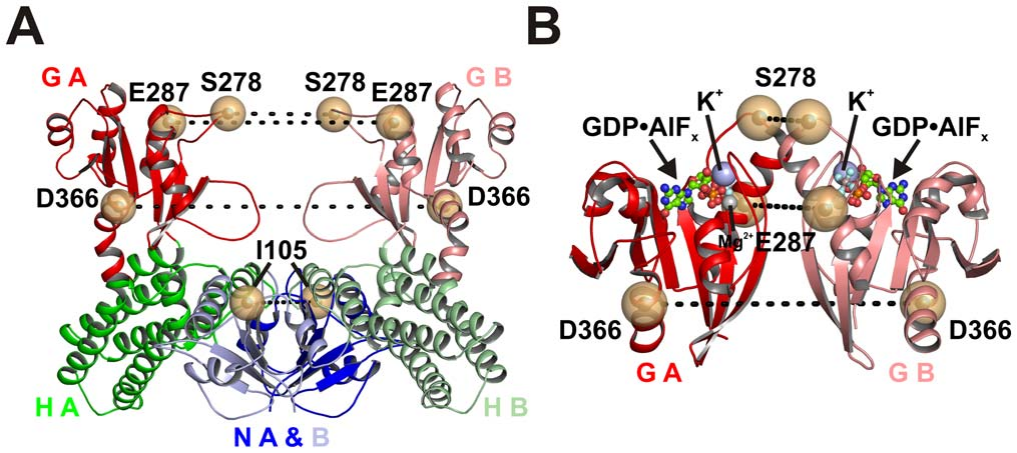
Spin label sites in the MnmE dimer. Position of residues that were mutated to Cys and spin labeled (yellow spheres), with dashed lines indicating distances between residues in the open (A) and closed (B) conformation. Domains are labeled as in Figure 1. In (B) GDP-AlF_x_ is displayed as stick model, Mg^2+^ as grey sphere and K^+^ as blue spheres. Cβ-Cβ distances were calculated from the respective residues for the open conformation represented from the model in Figure 1a (generated with pdb 1XZP) and the closed conformation obtained from the structures of the G domain in the GDP-AlFx state (pdb 2GJ8), as summarized in Table 2.

To test whether the “open” G domain arrangement found in our GDP- and GppCp-bound structures is representative for the conformation in solution and to identify and characterize the putative transition state with closed G domains, which could not be obtained by crystallization, we applied four-pulse DEER spectroscopy [36]–[38], to measure distances between nitroxide spin labels in the G domains of full-length EcMnmE in different steps of the GTPase cycle. Positions mutated to cysteine for spin labeling with (1-oxyl-2,2,5,5-tetramethyl-3-pyrroline-3-methyl) methanethiosulfonate spin label (MTSSL) are Glu287, close to the top of the G domain in Gα2, Ser278 in switch II, and Asp366, located in Gα6, and, as shown in Figure 3, result in the introduction of two symmetry-related spin labels in the functional MnmE dimer. As a possible “negative control” we also spin labeled position Ile105 in the N-terminal domain, for which no distance changes are expected. The Cβ-Cβ distances between these sites derived from the structures of the open and the model of closed state are listed in Table 2. To avoid unwanted side effects of cysteine substitutions, only nonconserved, surface-exposed residues have been selected. Furthermore, mutant proteins were assayed for K^+^-stimulated GTPase activity with and without attached MTSSL-label. No impairment of GTPase activity in comparison to wild type could be observed by the mutation itself or the introduction of the spin label (Table S1). Since efficient GTPase activity in the presence of K^+^ is strictly dependent on correct K^+^-binding and G domain dimerization [22], we can conclude that the structural and functional aspects of G domain dimerization and GTPase activity of the mutants are preserved in the proteins used for DEER.

**Table 2:** Cβ-Cβ distances between pair of residues mutated to Cys for MTSSL labeling measured in various MnmE dimer crystal structures and maxima in distance distributions for the pair of spin labels from experimentally determined DEER distance distributions.

Residue^a^ Mutated to Cys	Nucleotide State	Cβ-Cβ Distance from X-ray Structures/Å	Maximum in DEER Distance Distribution/Å^b^
E287R1, Gα2	apo	53^c^	55
	GDP	—	53
	GppNHp	—	37, 55
	GDP-AlF_x_	28^d^	36
E366R1, Gα6	apo	62^c^	67
	GDP	**57** e, 63^f^	65
	GppNHp	49^g^, 53^h^	47, 63
	GDP-AlF_x_	47^d^	**48**, 58
S278R1, switch II	apo	22^c^	25–50 (**46**)
	GDP	—	25–50 (**47**)
	GppNHp	—	27, **43**
	GDP-AlF_x_	18^d^	28
I105R1, N-terminal domain	apo	37^c^	29
	GDP	36^e^, 37^f^	—
	GppNHp	36^g^, 36^h^	—
	GDP-AlF_x_	—	29

Note that not all residues selected for spin labeling are resolved in all X-ray structures.

a Numbering according to *E. coli* MnmE sequence.

b Major maxima are highlighted in bold.

c *T. maritima* homodimer model (generated with pdb 1XZP).

d From *E. coli* G domain dimer (pdb 2GJ8).

e From CtMnmE·GDP.

f From NoMnmE·GDP.

g From CtMnmE·GppCp, dimer A.

h From CtMnmE·GppCp, dimer B.

### Nucleotide Free and GDP-Bound State

**Figure 4:**
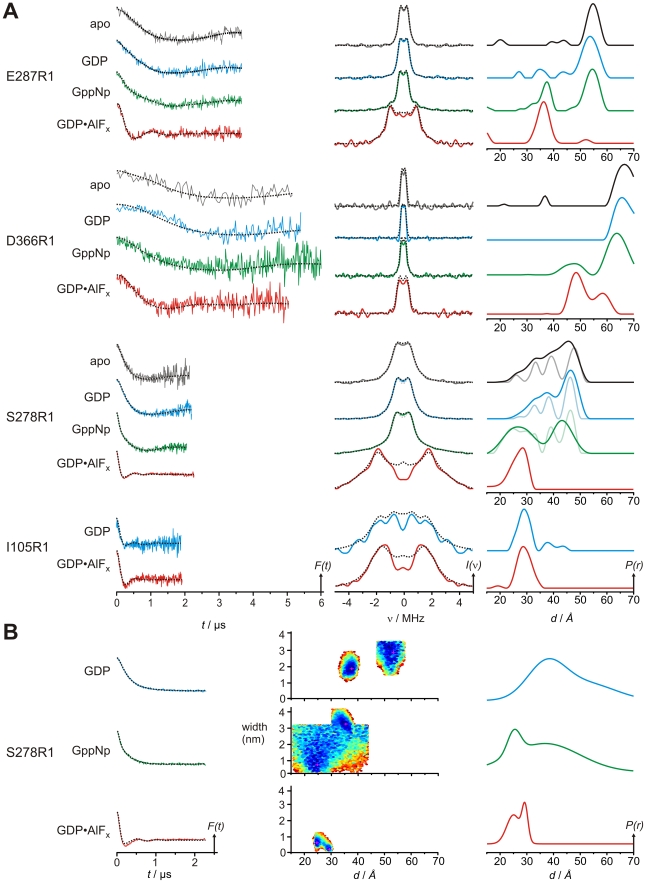
DEER characterization of nucleotide-dependent domain movements of MTSSL labeled MnmE (E287R1, D366R1, S278R1, and I105R1). (A) Left panel, background corrected dipolar evolution data for the apo, GDP, GppNHp, and GDP-AlF_x_ state of the respective MnmE mutants as indicated. Centre panel: dipolar spectra (Fourier transformation of the dipolar evolution data in the left panel). Right column: distance distributions obtained by Tikhonov regularization. All plots are normalized by amplitude. Broken lines in the left and center panel are fits to the data obtained by Tikhonov regularization. For S278R1 in apo, GDP, and GDP-AlF_x_ state, alternative fits and resulting distance distributions obtained with smaller regularization parameters α, are shown in corresponding pale colours. (B) Data for S278R1 in the GDP-, GppNHp-, and GDP-AlF_x_ state analyzed assuming a sum of Gaussian distributed conformers. Left panel: background corrected dipolar evolution data. Centre panel: goodness-of-fit (χ^2^) surfaces, created by random sampling of distance and width for each Gaussian population in the distance distributions shown in the right panel. Plots in the left and right column are normalized by amplitude. Broken lines in the left panel are fits to the data.

Figure 4A illustrates the results of the DEER measurements in the presence of 100 mM KCl, where the left panel shows the background-corrected dipolar evolution data, the centre panel the respective dipolar spectra, and the right panel the corresponding distance distributions (obtained by Tikhonov regularization; see Methods), which are summarized in Table 2. The DEER analysis of mutant E287R1 (R1 denotes the MTSSL side chain), close to the top of the G domain in Gα2, indicates one major peak centered at a distance of 55 Å for the apo- and 53 Å for the GDP-bound state. This distances correspond well to the Cβ-Cβ distances in the TmMnmE crystal structure model of 53 Å (the corresponding residues in the CtMnmE and NoMnmE structures are not resolved) and is therefore in agreement with an open conformation of the G domains. For D366R1 (situated at Gα6), a well-defined interspin distance distribution centered at 67 Å in the apo- state and 65 Å in the GDP-bound state could be observed in good agreement with the distances obtained from the TmMnmE dimer model (62 Å) and NoMnmE·GDP (63 Å), suggesting again an open conformation of the G domains. The corresponding Cβ-Cβ distance in CtMnmE·GDP dimer is somewhat shorter (57 Å), which is due to the different orientation of G domains in this structure (Figure 2A) and to the different tilting of Gα6 (Figure 2B). From the E287R1 and D366R1 data in the apo- and GDP-bound states, we conclude that instead of a continuum of freely moving orientations, the MnmE G domains seem to have defined major orientation reflected by the distance distributions.

In contrast, the analysis for S278R1 (switch II region) by Tikhonov regularization did not allow discrimination between a continuum of distances ranging from 25 Å to 50 Å with increasing probabilities for larger distances (shown in dark colours) or three to four distinct distances corresponding to different protein and/or spin label conformers (shown in pale colours). To clarify this issue, we additionally fitted the GDP data with a Monte Carlo/SIMPLEX algorithm assuming a sum of Gaussian-distributed conformers contributing to the dipolar evolution data (Figure 4B) [41]. The experimental data were satisfactorily reproduced by a distance distribution with two Gaussian populations, which are well defined as judged by the χ^2^ surfaces, summing up to a broad distribution in the range 30–50 Å. Possible explanations for such a continuum in the distance distribution could be (i) that the labeled position is located in the switch II region, which is flexible in the free and GDP-bound states, in line with the X-ray results, or (ii) that the spin label side chains are not restricted in their conformational space and populate multiple rotamers, or (iii) a combination of (i) and (ii). A general continuum of G domain orientations can be excluded from the results for positions E287R1 and D366R1. Control measurements of K^+^-stimulated GTPase activity (Table S1) make severe structural perturbations appear unlikely. Instead the deviation from the Cβ-Cβ distance of 22 Å in the TmMnmE dimer model is probably due to a switch II conformation induced by crystal packing forces. It has been observed before, that even in structures of the same G protein-nucleotide complex different switch II conformations were induced by crystal packing forces [42]. Nevertheless, the most pronounced distances between 40–50 Å as well as the minor fractions situated between 30 and 40 Å observed by DEER are in strong agreement with an open state of the G domains as observed in the apo- and GDP-bound crystal structures.

### GppNHp-Bound State

In the presence of the nonhydrolizable GTP analogue guanosine 5′-imidotriphosphate (GppNHp) the distance distributions comprise two fractions with different interspin distances for all three labeled positions. One larger distance (E287R1, 55 Å; D366R1, 63 Å; and S278R1, 43 Å) corresponds to the open state of the G domains as observed for the nucleotide-free and GDP-bound forms, whereas the other distance, contributing about 30% to the distance distribution (average value calculated from the area under the distance distribution curve) is characterized by significantly shorter distances (E287R1, 37 Å; D366R1, 47 Å; and S278R1, 27 Å), clearly indicating the presence of a second conformation, where the two G domains are in close proximity. As for the GDP-bound state, the GppNHp data for S278R1 were additionally fitted assuming a sum of Gaussian distributions. Despite differences especially in the distribution width for the two populations, this approach also reveals the presence of the two conformations of the G domains. In the X-ray structure of the AlF_x_-complexed G domain dimer, the Cβ-Cβ distance of the S278- and E287-pair are 18 Å and 28 Å, respectively and thus somewhat shorter as compared to the GppNHp DEER data (S278R1, 27 Å; E287R1, 37 Å). However the MTSSL-side chain itself has an average length of 7 Å between the nitroxyl-radical and the Cβ-atom [43]. This can increase the measured distance up to 14 Å for a pair of MTSSL side chains, depending on their rotamer orientation. The longer distances of the short distance maxima in the GppNHp-distance distributions of S278R1 and E287R1 measured in solution are thus most likely the result of a closed conformation of G domains, where the MTSSL side chains protrude away from the symmetry axis of the G domain dimer. Overall, the GppNHp measurements lead us to conclude that in the presence of GppNHp two conformations are in thermal equilibrium. In the crystal structure of GppCp-bound MnmE the G domains are found in the open state, indicating that this equilibrium is shifted towards the open state under the crystallization conditions.

### GDP-AlF_x_–Bound State

In the presence of the transition state mimic GDP-AlF_x_, S278R1 and E287R1 show a single population maximum, with defined distances of 28 Å and 36 Å, respectively, in line with a closed conformation (Figure 4). The observed distances are close to the observed Cβ-Cβ distances in the crystal structure of the GDP-AlF_x_–bound G domain dimer structure (S278, 18 Å; E287 28 Å), with deviations due to spin label conformations as discussed above. Compared to the distances characterizing the closed conformation in the presence of GppNHp, the distance distributions for the transition state mimic are sharper and the maxima are slightly shifted. For E287R1 it decreases by about 1–2 Å and for position S278R1 the broad distribution between 20 and 30 Å converts to a more defined but asymmetric distribution with a major distance of 28 Å, which is well reproduced also by the Monte Carlo approach (Figure 4B). For position D366R1 two major fractions with inter spin distances of 58 and 48 Å are visible, presumably due to two different rotamer populations of the spin label side chain. The maximum at 48 Å corresponds nicely to the Cβ-Cβ distance in the GDP-AlF_x_–bound G domain structure, whereas the 58Å distance likely represents an MTSSL-rotamer population pointing away from each other. As is obvious from the distance distributions for the GppNHp and the GDP-AlF_x_ state, the closed state in the presence of GDP-AlF_x_ slightly differs from that in the presence of GppNHp suggesting that on the reaction pathway from the triphosphate state to the GTPase competent conformation further rearrangements in the active site of the G domains take place. Overall the distance maxima are shifted to shorter distances in the GDP-AlF_x_ state as compared to the apo-, GDP- and GppNHp distances. This shows that the G domains adapt a closed conformation as observed in the GDP-AlF_x_-complexed G domain structure.

### Position Ile105 in the N-Terminal Domain

To explore whether G domain dimerisation leads to domain rearrangements in the N-terminal dimerization domain, a spin label was introduced at position Ile105 (Figure 3A). A comparison of the distance distributions obtained for the GDP state (open conformation) and GDP-AlF_x_ state (closed conformation) does not show any significant differences concerning the major population in the distance distribution with an average distance of 29 Å for both nucleotide states (Figure 4A; Table 2), indicating, that closing of the G domains does not significantly disturb the overall integrity of the N-terminal domains. The deviation to the corresponding Cβ-Cβ distances in the various dimer models (36 Å, 37 Å) are likely due to spin label rotamer conformations.

### Cation Dependence of G Domain Dimerization

**Figure 5:**
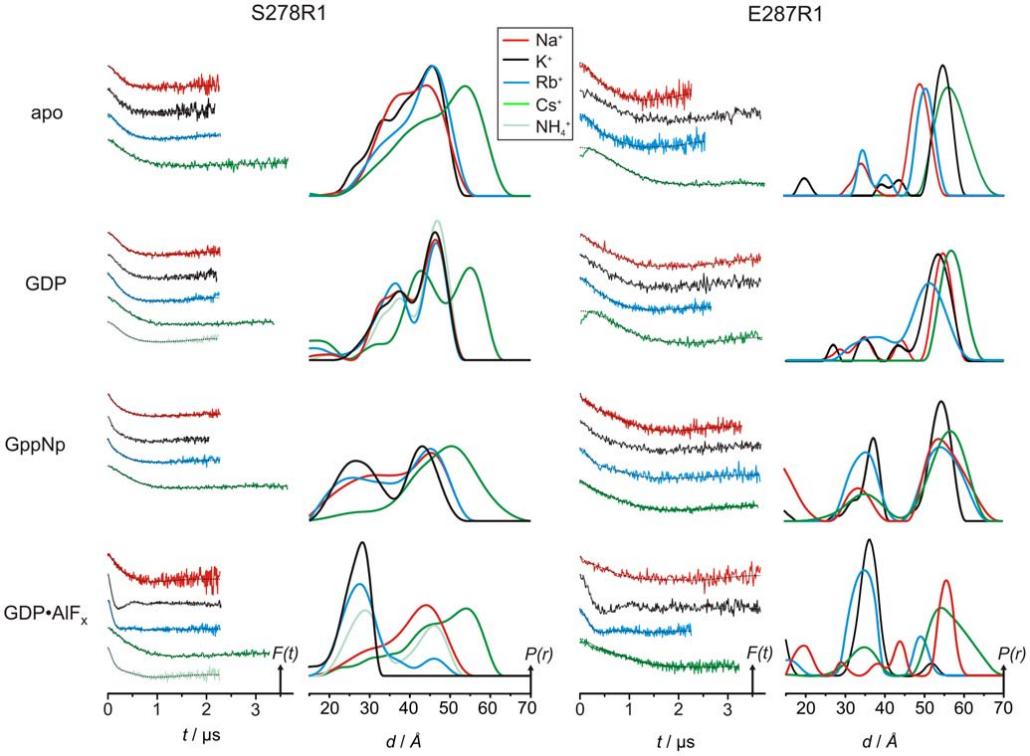
Cation dependency of DEER distance distributions, for MnmE mutants S278R1 (left) and E287R1 (right). For each mutant, the left column shows the background corrected dipolar evolution data and the fit obtained by Tikhonov regularization (broken line) and the right column the corresponding distance distribution. The evolution data and the respective distance distributions are colored according to the cation present in the experiment (red, Na^+^; black, K^+^; blue, Rb^+^; green, Cs^+^; and pale green, NH_4_^+^ [only for S278R1, GDP, and GDP-AlF_x_]). The area under the distance distribution corresponds to the number of interacting spins, derived from the modulation amplitude of the background corrected dipolar evolution data.

Previous studies have shown K^+^ ions to activate the MnmE GTPase. This follows from the finding that dimerization of the MnmE G domains and GDP-AlF_x_ complex formation strictly require K^+^, which is bound in the dimer interface (Figure 3B), such that its position overlaps with that of an Arg finger required for the GAP-mediated GTP hydrolysis on Ras-like G proteins [20],[22]. Moreover, GTPase activity and AlF_x_-induced dimerization are at least partially stimulated by cations with an ionic radius comparable to K^+^ (1.38 Å) such as Rb^+^ (1.52 Å) and, to a lesser extent, NH_4_^+^ (1.44 Å), whereas Na^+^ (0.99 Å) and Cs^+^ (1.67 Å) do not show this effect [22]. Consistent with this, Rb^+^ and NH_4_^+^ were also found to be coordinated to the K^+^ binding site in two MnmE G domain dimer structures GDP complexed with AlF_x_ (pdb 2GJ9 and 2GJA) [22]. To analyze the cation dependency of G domain dimerization in full-length protein in solution, we determined distance distributions for the sites S278R1 and E287R1 in the apo, GDP, GDP-AlF_x_, and GppNHp bound state in the presence of various cations, i.e., Na^+^, K^+^, Rb^+^, Cs^+^, and for S278R1 additionally in the presence of NH_4_^+^ for the GDP and GDP-AlF_x_ state (Figure 5).

The distance distributions and dipolar time traces show that in the presence of GDP-AlF_x_ only K^+^ is capable for shifting the equilibrium completely towards the closed G domain dimer. The ability of the respective cations to stabilize G domain dimerization follows the order K^+^>Rb^+^>NH4^+^>Cs^+^≈Na^+^, clearly correlated with their ionic radii and their ability to stimulate GTP hydrolysis [22]. In the presence of GppNHp, we observe the same order of cations with regard to their capability for shifting the equilibrium towards the closed state. Notably, Cs^+^, which is completely unable to stabilize G domain dimerization, seems to have an influence on switch II conformational dynamics and on the overall orientation of the G domains, as seen from the significantly broadened and shifted distance distributions compared to those for the other cations.

## Discussion

Understanding how GADs use the GTPase cycle as the driving force to perform a variety of functions like insertion of signal sequences into the ER translocon by the SRP/SR system [44], tRNA modification by MnmE [19],[22], kinase activation by the Parkinson kinase LRRK2 [45], or metal ion delivery to hydrogenases [46] is a crucial step for elucidating the diverse mechanism by which these proteins operate. Although within this class of proteins MnmE is one of the structurally and biochemically best characterized and a model for the GTPase cycle dependent G domain dimerization has been proposed [22], neither the structural model of the full-length MnmE dimer nor dimerization of the G domains in the full-length dimeric protein have been proven directly.

Here we have applied a combined approach of X-ray crystallography and pulse electron paramagnetic resonance (EPR) spectroscopy to study the behavior of the G domains in full-length MnmE in different steps of the GTPase cycle. We were able to solve the first X-ray structures of full-length MnmE in complex with nucleotides. The structures confirm the previously postulated homodimer model [19] according to which MnmE constitutively dimerizes via its N-terminal domain whereas the G domains, separated by a large distance of approximately 48 Å (measured from Cα of the first glycine of the P-loop), face each other with their nucleotide binding sites. The distance distributions obtained by DEER of MnmE in the apo, GDP, and GppNHp state reveal that the G domains are far apart also in solution excluding that the open conformations in the crystal structures are crystallographic artefacts. Comparison of the different full-length structures reveals that the G domains are present in drastically different orientations suggesting them to be highly mobile elements capable of moving independently with regard to the other domains. As judged from the X-ray structure, they need to overcome a 20–30-Å distance gap on formation of the GDP-AlF_x_ complex [22]. In contrast to the X-ray data, the DEER distance distributions suggest the presence of one defined orientation for the open state in solution, arguing that the different G domain orientations in the X-ray structures result from crystal packing forces. That reasonable diffraction data can only be obtained when the G domains are stabilized by packing interaction is a further indication for their high mobility. Moreover, for CtMnmE we find different orientations between the GDP- and GppCp-bound structures and even between different molecules in one asymmetric unit of CtMnmE·GppCp.

Although the crystals for all structures presented here were grown in the presence of K^+^ and triphosphate or a transition state mimic to induce G domain dimerization, the structures show the G domains in an open state, suggesting that the closed state is not stable under crystallization conditions. We can demonstrate indeed using a fluorometric assay with mant-GDP, that close juxtaposition of G domains with AlFx is inhibited in the presence of crystallization precipitants.

The interspin distances between the spin labeled G domains obtained by DEER directly prove for the first time that the G domains contact each other in the presence of triphosphate or transition state analogs. A notable feature of the GppNHp-bound state is the coexistence of an open and closed state, pointing out that a triphosphate analog is not sufficient to fully stabilize the closed state. However, recently a stabilizing effect of GidA on the closed state of the G domains was shown, indicating that regulation of the MnmE G domain cycle is coupled to other components of the tRNA-modification system [6]. Unlike the results from X-ray structures, the EPR data, under low salt and in the absence of PEG, do not show a continuum of conformations but rather particular conformations in the open and closed state not observable in the X-ray experiment. We further show that only the presence of GDP, AlF_x_ and K^+^ is capable of stabilizing the closed state, and that this effect is specific, since the effect is absent with Na^+^ and Cs^+^ and is smaller with similar size cations such as Rb^+^ and NH_4_^+^.

In summary, we were able to directly demonstrate the conformational changes MnmE was suggested to undergo during its GTPase cycle [6],[19],[22],[39],[47]. Dimerization of the MnmE G domains is accompanied by large domain movements of up to 20 Å from the open to the closed state, which is an apparently unique feature of MnmE with regard to other GADs, suggestive for a functional or regulatory coupling of these domain movements to the tRNA-modification reaction. For the architecturally similar Roc-COR tandem (see above), the G domains in the nucleotide free state are already in close proximity [35], rendering similar extensive domain rearrangements unlikely. Yet such drastic rearrangements are not untypical for NTPases, as for example Hsp90, which constitutively dimerizes via its C-terminal domain, undergoes dramatic domain movements during its ATPase cycle involving juxtaposition of its N-domains in the triphosphate state [48],[49].

MnmE forms a heterotetrameric complex with GidA [50], which is stabilized in the triphosphate state [6],[39], and tRNA modification was suggested to be exerted by this complex rather than by the individual proteins [5],[50], which was recently proven by an in vitro modification assay [6]. Furthermore active GTP-turnover rather than simple GTP-binding was shown to be essential for the modification reaction [6],[47] and in particular, nucleotide dependent G domain dimerization is tightly coupled to the tRNA-modification process both in vitro and in vivo [6]. According to a proposed reaction mechanism, the reaction itself does not require energy, but rather comprises several steps at presumably different, spatially separated active sites, requiring tight regulation [19],[39]. We thus speculate that G domain dimerization during GTP hydrolysis is required for orchestration of the multistep tRNA-modification reaction [6]. The exact link between G domain dimerization, GTP hydrolysis, conformational changes, and tRNA modification is focus of current investigations.

## Materials and Methods

### Proteins

*C. tepidum* and *Nostoc* sp. 7120 MnmE (CtMnmE, NoMnmE) were cloned into pET14b (Novagene) and expressed as N-terminal His-tagged proteins in *Escherichia coli* BL21-DE3. Cells were lysed in 50 mM Tris (pH 7.5), 100 mM NaCl, 5 mM MgCl_2_ ( = buffer A) with 20 mM imidazole, 5 mM β-mercaptoethanol, 150 µM PMSF, and the proteins were purified by Ni-NTA, thrombin-cleavage of the His-Tag, and gel filtration on Superdex 200 in buffer A with 5 mM dithioerythritol (DTE). Cloning, expression, and purification of *E. coli* MnmE and mutants and preparation of nucleotide-free MnmE was carried out as described elsewhere [39].

### Crystallography

Crystals were obtained by hanging-drop vapour diffusion. For CtMnmE·GDP crystals, 1 µl each of 50 mg/ml protein in 50 mM Tris (pH 7.5), 100 mM KCl, 5 mM MgCl_2_, 5 mM DTE (buffer B) plus 5 mM GDP, 5 mM AlCl_3_, 50 mM NaF, and precipitant (100 mM Tris-HCl [pH 8.5], 2.250 M NaCl, 15% [w/v] PEG 6000) were mixed. After 3 d the reservoir was changed to 100 mM Tris-HCl (pH 8.5), 2.250 M NaCl, 30% PEG 6000, and equilibrated for 2 more days. Crystals were soaked with precipitant supplemented with 12% glycerol and 5 mM 5-F-THF for 30 min and flash-frozen in liquid nitrogen. For NoMnmE·GDP, 1 µl of 20 mg/ml protein in buffer B with 5 mM GDP, 5 mM AlCl_3_, 50 mM NaF, and precipitant (100 mM Tris [pH 7.5], 22% [w/v] PEG 550 MME, 10 mM ZnSO_4_) were mixed and grown at 20°C. After 2 d crystals were cryo-dipped into reservoir solution with 28% (w/v) PEG 550 MME and flash-frozen into liquid nitrogen. For CtMnmE·GppCp, 40 mg/ml nucleotide free protein in buffer B with 5 mM GppCp was mixed (1∶1) with 100 mM MES, 46 mM NaOH, 12% PEG 4000, 40 mM NaCl, and crystals were grown at 20°C. After 2–3 d, crystals were flash-frozen in reservoir containing 20% glycerol. All datasets were collected at 100 K on beamline PX2 (SLS, Villingen) at wavelengths of 0.98003, 0.9796, and 1.28186 Å (Zn^2+^-edge) for CtMnmE·GDP, CtMnmE·GppCp, and NoMnmE·GDP, respectively. All datasets were processed, indexed, and scaled with XDS [51].

Initial phases were obtained by molecular replacement with the N-terminal and the helical domain of *T. maritima* MnmE (pdb 1XZP) with MOLREP [52]. Coot [53] and REFMAC [54],[55] were used for model building and translation, libration, screw rotation (TLS)-refinement including NCS restraints and NCS-averaged maps in the case of CtMnmE·GppCp. Crystallographic simulated annealing of models was carried out with CNS [56]. Structural representations were prepared with pymol (www.pymol.org). For NoMnmE·GDP, Zn^2+^ atom positions were located by their anomalous signal. For CtMnmE·GppCp, a positive peak in the F_O_-F_C_-map close to the β- and γ-phosphate in the nucleotide binding site of G domain A was assigned to Mg^2+^, on the basis of its position at the usual Mg^2+^-site in G protein structures. Structures were analyzed by PROCHECK [57] revealing for all three structures 100% of torsion angles within the allowed Ramachandran regions. Data collection and refinement statistics are listed in Table 3. Structures were aligned with coot [53] and Superpose of the CCP4-package [54]. Crystal contact areas were calculated using the PROTORP server [58].

**Table 3:** Data collection and refinement statistics.

Name	CtMnmE·GDP^a^	CtMnmE·GppCp^a^	NoMnmE·GDP^a^
Pdb-code	3GEE	3GEI	3GEH
Data collection			
Dataset type	Native	Native	Native
Space group	I4(1)22	C222(1)	P4(3)2(1)2
Cell dimensions			
*A*, *b*, *c* (Å)	130.804, 130.804, 200.611	139.882, 224.572, 156.788	124.279, 124.279, 174.701
α, β, γ (°)	90.0, 90.0, 90.0	90.0, 90.0, 90.0	90.0, 90.0, 90.0
Resolution (Å)	20.00−2.95 (3.00−2.95)	20.00−3.40 (3.42−3.40)	20.00−3.20 (3.30−3.20)
*R*_merge_	7.7 (71.7)	12.9 (65.4)	12.7 (48.5)
*I*/σ**	24.49 (2.39)	12.13 (2.02)	8.61 (2.18)
Completeness (%)	98.9 (99.7)	99.2 (100.0)	99.4 (99.9)
Redundancy	7.16 (7.28)	7.45 (7.61)	7.78 (8.03)
Refinement			
Resolution (Å)	19.90−2.95 (3.03−2.95)	19.94−3.40 (3.49−3.40)	20.00−3.20 (3.28−3.20)
*n* Reflections	17,528	32,320	21,913
*R*_work_/*R*_free_	0.23/0.27	0.24/0.27	0.24/0.27
*n* Atoms	3,321	8,780	3,428
Protein	3,259	8,715	3,364
Ligand/Ion	62	65	64
*B*-factors (Å^2^)	106.21	123.56	52.69
Protein	105.96	124.35	51.85
Ligand/Ion	115.84	164.78	97.10
Root mean square deviations			
Bond lengths (Å)	0.006	0.008	0.007
Bond angles (°)	1.147	1.198	1.185

Values in parentheses are for the highest-resolution shell.

a Data from one crystal.

### Fluorometric Detection of AlF_x_-Complex Formation

10 µM of nucleotide-free *E. coli* MnmE loaded with 0.5 µM of mGDP were incubated in 50 mM TriS-HCl (pH 7.5), 100 mM KCl (or NaCl), 5 mM MgCl_2_, 10 mM NaF with or without the precipitants 15% PEG 6000, 2,250 mM NaCl, or both or 22% PEG 550 MME at 20°C. The fluorescence of mGDP bound to MnmE, excited at 366 nm and detected at 450 nm, was monitored over time in a Fluoromax 2 spectralfluorimeter (Spex Industries). To initiate AlF_x_-complex formation, 1 mM AlCl_3_ was added and the fluorescence was continuously monitored. For analysis, fluorescence amplitudes were normalized to the amplitude before addition of AlCl_3_.

### Spin Labeling

Purified, nucleotide-free Cys-mutants of *E. coli* MnmE-C451S (Table 2) were pretreated with DTE (4°C). After removal of DTE protein solutions were incubated with 1–5 mM MTSSL (Toronto Research, Alexis) for 16 h (4°C). Excess MTSSL was removed by gel filtration. Labeling efficiencies have been determined to be >80% in all cases.

### Steady State GTPase Measurements

GTPase reactions were started by adding 0.5 µM of wild type or mutant MTSSL-labeled or nonlabelled MnmE protein to 186 µM of GTP in 50 mM Tris-HCl (pH 7.5), 100 mM KCl, 5 mM MgCl_2_, and performed at 20°C. At time points 0, 1, 2, 3, 5, 7, and 10 min aliquots were taken and analyzed for their nucleotide content by HPLC as described elsewhere [22]. For comparison, *v*_app_ was determined as the absolute value of the slope of a linear fit of GTP consumption over time, normalized to the total amount of enzyme for a range in which 10% of initial GTP was consumed.

### Pulse EPR Measurements

Pulse EPR experiments (DEER) were accomplished at X-band frequencies (9.3–9.4 GHz) with a Bruker Elexsys 580 spectrometer equipped with a Bruker Flexline split-ring resonator ER 4118X-MS3 and a continuous flow helium cryostat (ESR900, Oxford Instruments) controlled by an Oxford Intelligent temperature controller ITC 503S. Buffer conditions for the EPR experiments were 200–500 µM protein in 100 mM KCl (or NaCl, RbCl, CsCl, NH_4_Cl), 50 mM Tris-HCl, 5 mM MgCl_2_ (pH 7.4) with 5% (v/v) ethylene glycol (for H_2_O buffer) or 12.5% (v/v) glycerol-d_8_ (for D_2_O buffer), and 1 mM GDP, 1 mM GppNHp or 1 mM GDP, 1 mM AlCl_3_, 10 mM NaF, respectively.

All measurements were performed using the four-pulse DEER sequence: 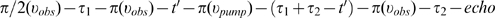 [59]. A two-step phase cycling (+ 〈x〉, − 〈x〉) was performed on 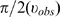. Time *t′* is varied, whereas τ_1_ and τ_2_ are kept constant, and the dipolar evolution time is given by 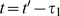. Data were analyzed only for *t*>0. The resonator was overcoupled to Q∼100; the pump frequency υ_pump_ was set to the center of the resonator dip and coincided with the maximum of the nitroxide EPR spectrum, whereas the observer frequency υ_obs_ was 65 MHz higher, coinciding with the low field local maximum of the spectrum. All measurements were performed at a temperature of 50 K with observer pulse lengths of 16 ns for π/2 and 32 ns for π pulses and a pump pulse length of 12 ns. Proton modulation was averaged by adding traces at eight different τ_1_ values, starting at 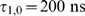 and incrementing by 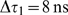. For proteins in D_2_O buffer with deuterated glycerol used for their effect on the phase relaxation, corresponding values were 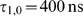 and 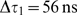. Data points were collected in 8-ns time steps or, if the absence of fractions in the distance distribution below an appropriate threshold was checked experimentally, in 16- or 32-ns time steps. The total measurement time for each sample was 4–24 h. Analysis of the data was performed with DeerAnalysis2006.1/2008 [60]. Additionally, the data was fitted assuming a sum of Gaussian distributed conformers utilizing the program DEFit 3.9 [41], which employs a Monte Carlo/SIMPLEX algorithm to find a distance distribution to which the corresponding dipolar evolution function represents the best fit to the experimental data.

### Accession Codes

Protein Data Bank (PDB) (http://www.rcsb.org/pdb): Coordinates und structure factors have been deposited with accession codes 3GEE (CtMnmE·GDP), 3GEI (CtMnmE·GppCp), and 3GEH (NoMnmE·GDP).

## Supporting Information

Figure S1 **Ligand binding in MnmE X-ray structures.** Protein backbones are displayed as ribbons with the N-terminal domain, helical domain, and G domain colored in blue, green, and white, respectively. Ligands are shown as stick models, metal ions as blue spheres, and electron density maps as meshes. (A) Stereo image of the N-terminal domains of NoMnmE·GDP with the two bound 5-F-THF molecules and the 2F_O_-F_C_-map contoured at 2σ around the 5-F-THFs. (B) N-terminal domains of CtMnmE·GDP with the two bound 5-F-THF molecules and the 2F_O_-F_C_-map contoured at 2σ around the 5-F-THFs. (C–E) The bound nucleotide in the G domain of the structures CtMnmE·GDP (C), NoMnmE·GDP (D), and CtMnmE·GppCp (E) with the P-loop, the G-4-, and the G-5-mofiv [42] highlighted in red and with the nucleotide-F_O_-F_C_-omit-maps as green meshes, contoured at 3σ (C, E) and 4σ (D). (C) GDP-bound to CtMnmE. (D) GDP bound to NoMnmE with the Zn^2+^-ion and its anomalous map contoured at 3σ (purple mesh). (E) GppCp bound to CtMnmE. Additionally the F_O_-F_C_-map at the β- and γ-phosphate calculated after fitting in GppCp, contoured at 2.5σ (purple mesh) is shown. On the basis of structural knowledge of the nucleotide binding site of G proteins, this peak in the F_O_-F_C_-map was assigned to Mg^2+^.
(6.11 MB TIF)

Figure S2 **Fluorometric assessment of G domain dimerization upon AlF_x_-complex formation in the presence of precipitants used for crystallization**. Normalized fluorescence amplitudes as a functions of time of the fluorescence labeled GDP analogon mGDP bound to MnmE in the presence of K^+^ as positive control (red curve) or Na^+^ as negative control (black curve) and NaF plus the respective precipitants together with K^+^, as indicated. At the beginning of the gap in the time traces, AlCl_3_ was added to initiate AlF_x_-complex formation and G domain dimerization, which only occurs in the presence of K^+^ and leads to an increase in fluorescence and which is impaired in the presence of various precipitants and K^+^ or when K^+^ is replaced by Na^+^.
(1.29 MB TIF)

Figure S3 **Different orientations of the G domains.** Superimposition of the five available MnmE homodimer structures CtMnmE·GDP, NoMnmE·GDP, CtMnmE·GppCp dimer A and dimer B, *T. maritima* MnmE dimer model generated with pdb 1XZP (TmMnmE) in ribbon representation with the N-terminal and helical domains in grey and the G domains (G A, G B) colored according to legend.
(5.16 MB TIF)

Figure S4 **Stabilization of the G domains by crystal contacts.** (A) Section of the crystal lattice of NoMnmE·GDP with close-up inset of the Zn^2+^-ion involved in crystal packing. In the crystal packing interface of the G domain to the symmetry related molecule, a Zn^2+^-ion (shown as grey sphere with its anomalous density contoured at 3 σ displayed as green mesh), is complexed by side chain residues (shown as sticks) of the G domain and the helical domain of the symmetry related molecule. MnmE molecules are displayed as ribbon models in different colors. (B) Section of the crystal lattice of CtMnmE·GDP with MnmE molecules displayed as ribbon models in different colors. Two MnmE dimers are packed upside-down on each other with a toothing arrangement of the G domains.
(4.25 MB TIF)

Table S1 **Rates** ***v*** **_app_ of K^+^-stimulated GTP-hydrolysis for wild-type MnmE, nonlabelled and MTSSL-labelled (denoted with R1) mutant MnmE proteins.**
(0.03 MB DOC)

Video S1 **The mobile MnmE G domains.** The video shows sequentially the superimposed five available MnmE homodimer structures CtMnmE·GDP, NoMnmE·GDP, CtMnmE·GppCp dimers of molecules A and B, *T. maritima* MnmE dimer model generated with pdb 1XZP (TmMnmE) in ribbon representation with the N-terminal domains in blue, the helical domains in green and the G domains in red. The sequence in the video is: CtMnmE·GDP, CtMnmE·GppCp dimer A, CtMnmE·GppCp dimer B, TmMnmE, NoMnmE·GDP, TmMnmE, CtMnmE·GppCp dimer B, CtMnmE·GppCp dimer A. The video player may be set to playmode “infinite loop.”
(1.93 MB MOV)

## Abbreviations

5-F-THF: 5-formyl-tetrahydrofolate
AlF_x_: aluminium tri- or tetrafluoride
cmnm: carboxymethylaminomethyl
DEER: double electron-electron resonance
DTE: dithioerythritol
EPR: electron paramagnetic resonance
GAD: G protein activated by nucleotide-dependent dimerization
GAP: GTPase activating protein
GppCp: guanosine-5′-(β,γ-methylene)triphosphate
GppNHp: guanosine 5′-imidotriphosphate
mGDP: 2′-/3′-O-(N′-Methylanthraniloyl)-GDP
MME: monomethyl ether
MTSSL: (1-oxyl-2,2,5,5-tetramethyl-3-pyrroline-3-methyl) methanethiosulfonate spin label
PEG: polyethylene glycol
