# Supplementary figures and images for: Kissing G Domains of MnmE Monitored by X-Ray Crystallography and Pulse Electron Paramagnetic Resonance Spectroscopy
Source: PLoS Biol. 2009 Oct 6;7(10):e1000212. doi: 10.1371/journal.pbio.1000212 (PMC2749940; doi:10.1371/journal.pbio.1000212)

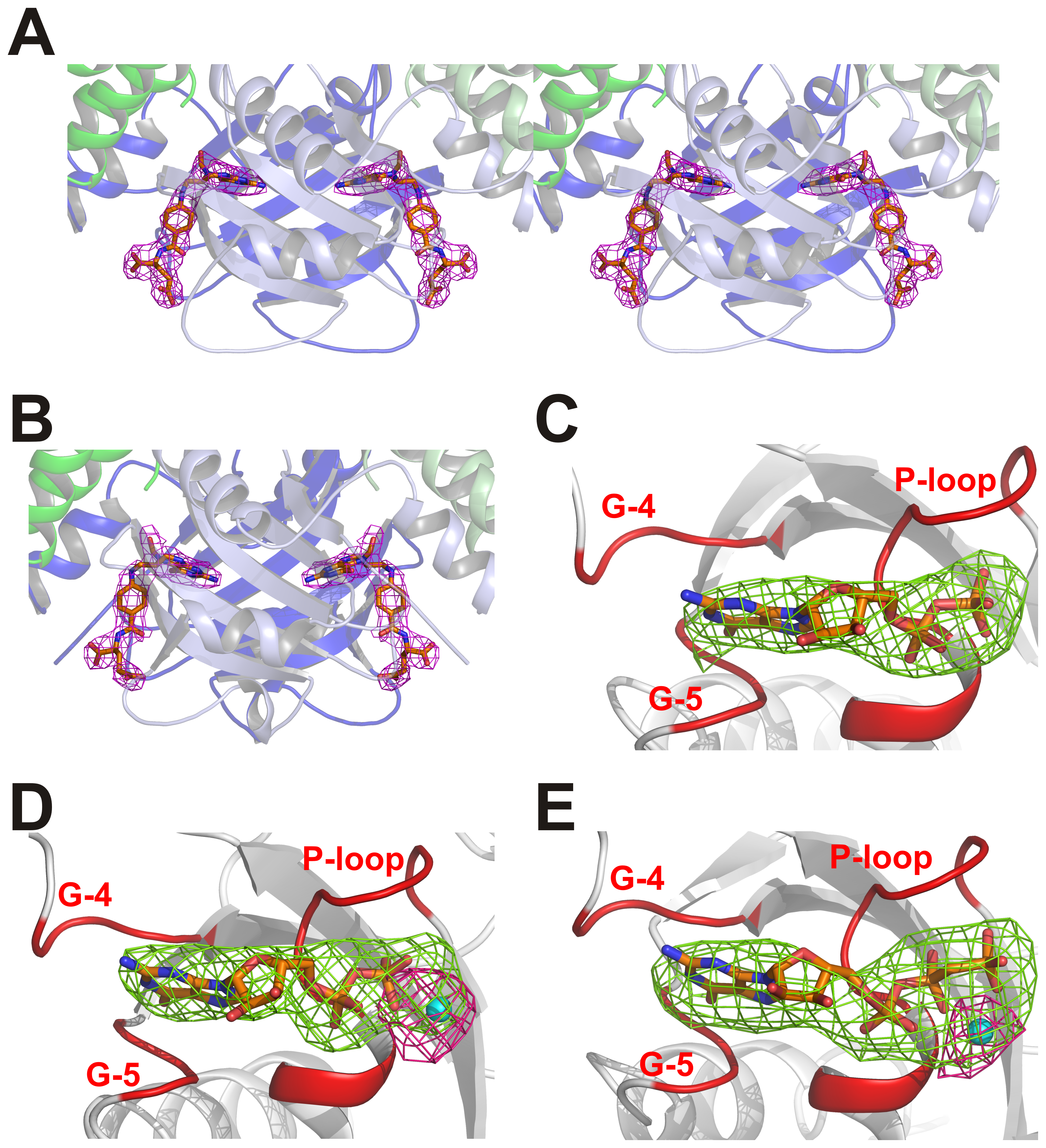

Supplement: Figure S1 — Ligand binding in MnmE X-ray structures. Protein backbones are displayed as ribbons with the N-terminal domain, helical domain, and G domain colored in blue, green, and white, respectively. Ligands are shown as stick models, metal ions as blue spheres, and electron density maps as meshes. (A) Stereo image of the N-terminal domains of NoMnmE·GDP with the two bound 5-F-THF molecules and the 2FO-FC-map contoured at 2σ around the 5-F-THFs. (B) N-terminal domains of CtMnmE·GDP with the two bound 5-F-THF molecules and the 2FO-FC-map contoured at 2σ around the 5-F-THFs. (C–E) The bound nucleotide in the G domain of the structures CtMnmE·GDP (C), NoMnmE·GDP (D), and CtMnmE·GppCp (E) with the P-loop, the G-4-, and the G-5-mofiv [42] highlighted in red and with the nucleotide-FO-FC-omit-maps as green meshes, contoured at 3σ (C, E) and 4σ (D). (C) GDP-bound to CtMnmE. (D) GDP bound to NoMnmE with the Zn2+-ion and its anomalous map contoured at 3σ (purple mesh). (E) GppCp bound to CtMnmE. Additionally the FO-FC-map at the β- and γ-phosphate calculated after fitting in GppCp, contoured at 2.5σ (purple mesh) is shown. On the basis of structural knowledge of the nucleotide binding site of G proteins, this peak in the FO-FC-map was assigned to Mg2+. (6.11 MB TIF) [file pbio.1000212.s001.tif]

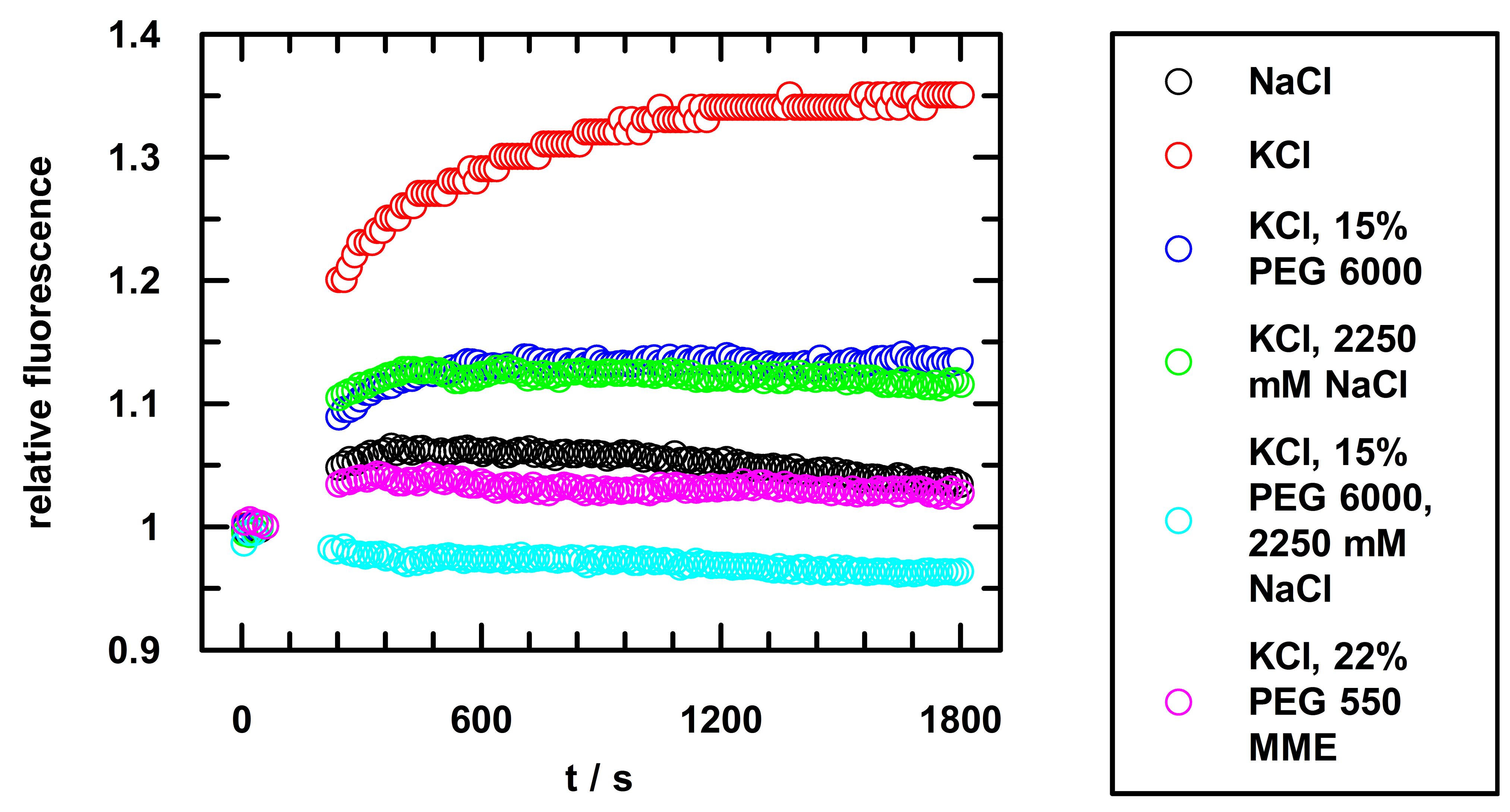

Supplement: Figure S2 — Fluorometric assessment of G domain dimerization upon AlFx-complex formation in the presence of precipitants used for crystallization. Normalized fluorescence amplitudes as a functions of time of the fluorescence labeled GDP analogon mGDP bound to MnmE in the presence of K+ as positive control (red curve) or Na+ as negative control (black curve) and NaF plus the respective precipitants together with K+, as indicated. At the beginning of the gap in the time traces, AlCl3 was added to initiate AlFx-complex formation and G domain dimerization, which only occurs in the presence of K+ and leads to an increase in fluorescence and which is impaired in the presence of various precipitants and K+ or when K+ is replaced by Na+. (1.29 MB TIF) [file pbio.1000212.s002.tif]

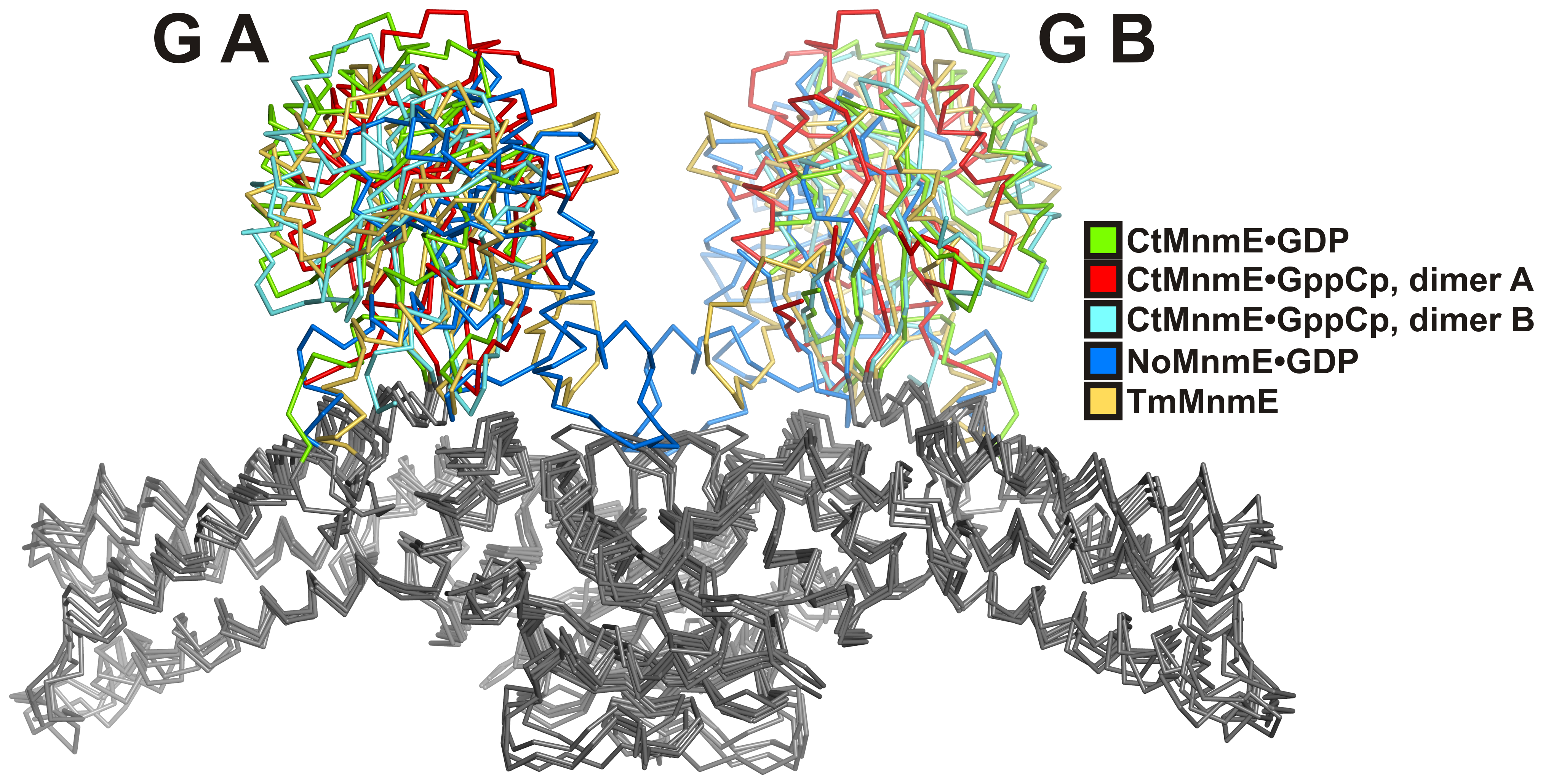

Supplement: Figure S3 — Different orientations of the G domains. Superimposition of the five available MnmE homodimer structures CtMnmE·GDP, NoMnmE·GDP, CtMnmE·GppCp dimer A and dimer B, T. maritima MnmE dimer model generated with pdb 1XZP (TmMnmE) in ribbon representation with the N-terminal and helical domains in grey and the G domains (G A, G B) colored according to legend. (5.16 MB TIF) [file pbio.1000212.s003.tif]

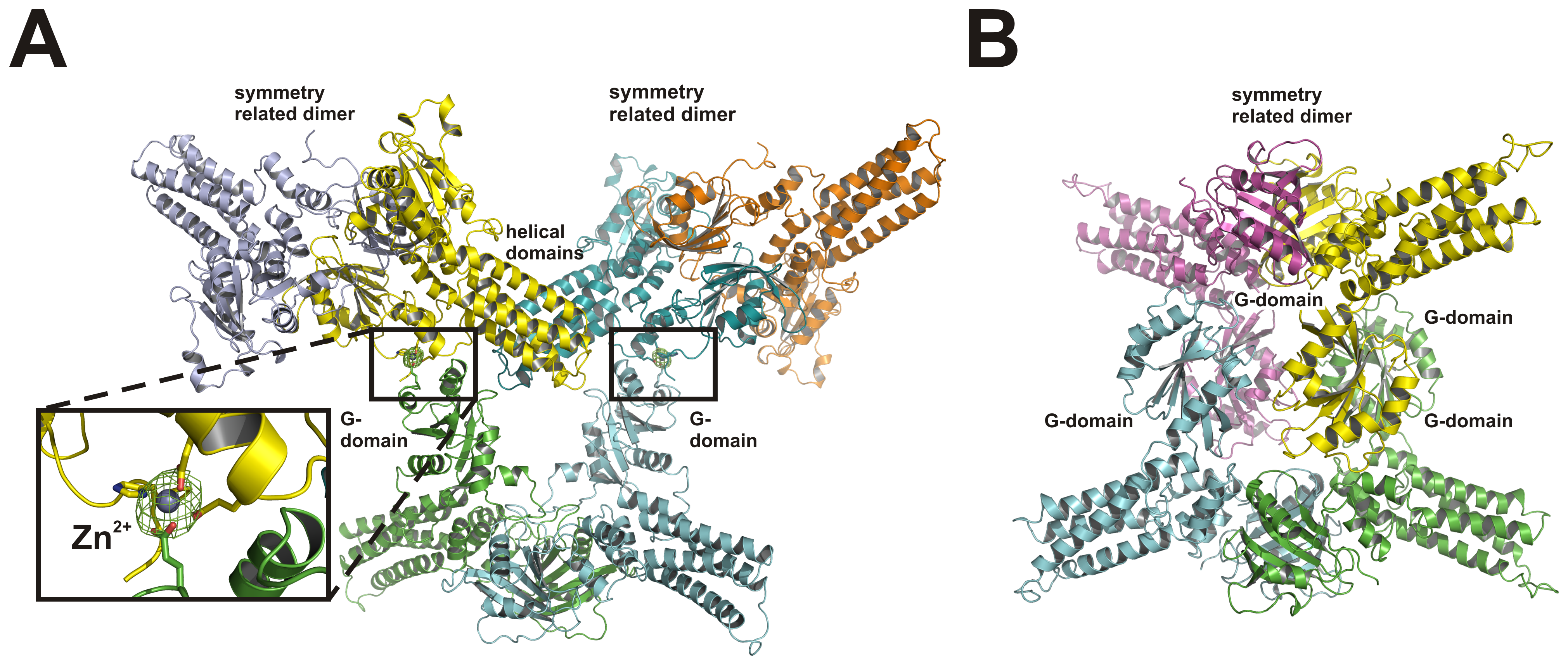

Supplement: Figure S4 — Stabilization of the G domains by crystal contacts. (A) Section of the crystal lattice of NoMnmE·GDP with close-up inset of the Zn2+-ion involved in crystal packing. In the crystal packing interface of the G domain to the symmetry related molecule, a Zn2+-ion (shown as grey sphere with its anomalous density contoured at 3 σ displayed as green mesh), is complexed by side chain residues (shown as sticks) of the G domain and the helical domain of the symmetry related molecule. MnmE molecules are displayed as ribbon models in different colors. (B) Section of the crystal lattice of CtMnmE·GDP with MnmE molecules displayed as ribbon models in different colors. Two MnmE dimers are packed upside-down on each other with a toothing arrangement of the G domains. (4.25 MB TIF) [file pbio.1000212.s004.tif]
