# Supplementary material for: Kissing G Domains of MnmE Monitored by X-Ray Crystallography and Pulse Electron Paramagnetic Resonance Spectroscopy
Source: PLoS Biol. 2009 Oct 6;7(10):e1000212. doi: 10.1371/journal.pbio.1000212 (PMC2749940; doi:10.1371/journal.pbio.1000212)
Supplement: Table S1 — Rates v app of K+-stimulated GTP-hydrolysis for wild-type MnmE, nonlabelled and MTSSL-labelled (denoted with R1) mutant MnmE proteins. (0.03 MB DOC) [file pbio.1000212.s005.doc]

**Table S1.** Rates *v_app_* ^1^ of K^+^-stimulated GTP-hydrolysis for wild type MnmE, non-labelled and MTSSL-labelled (denoted with R1) mutant MnmE proteins.

| Protein | *v_app_* / min^-1^ |
| --- | --- |
| MnmE wild type | 3.2 ± 0.2 |
| MnmE I105C | 3.0 ± 0.1 |
| MnmE I105R1 | 3.4 ± 0.1 |
| MnmE S278C | 4.2 ± 0.2 |
| MnmE S278R1 | 4.0 ± 0.1 |
| MnmE E287C | 3.0 ± 0.1 |
| MnmE E287R1 | 2.9 ± 0.1 |
| MnmE D366C | 2.8 ±.0.2 |
| MnmE D366R1 | 2.7 ± 0.1 |
| ^1^ *v_app_* is the absolute value of the slope of a linear fit of GTP consumption over time from multiple turnover experiments with 0.5 µM protein and 186 µM GTP for a range in which 10 % of initial GTP was consumed, normalized to the total amount of enzyme. | |
